# Gender equity in the scientific nursing journals indexed in Journal Citation Reports: A cross-sectional study

**DOI:** 10.3389/fpubh.2023.1119117

**Published:** 2023-03-17

**Authors:** Vicente Gea-Caballero, Regina Ruíz de Viñaspre-Hernández, Carlos Saus-Ortega, Luís Celda-Belinchón, Ivan Santolalla-Arnedo, Elena Marques-Sule, Raúl Juárez-Vela

**Affiliations:** ^1^Research Group in Community Health and Care (SALCOM), Faculty of Health Science, Valencian International University, Castelló de la Plana, Spain; ^2^Grupo de Investigación en Cuidados y Salud (GRUPAC), Faculty of Health Sciences, University of La Rioja, Logroño, La Rioja, Spain; ^3^Grupo de Investigación en Arte y Ciencia en Cuidados (GREAIC), Instituto de Investigación Sanitaria La Fe (IIS La Fe), Escuela Universitaria de Enfermería La Fe, Valencia, Spain; ^4^Physiotherapy in Motion, Multispeciality Research Group (PTinMOTION), Department of Physiotherapy, University of Valencia, Valencia, Spain

**Keywords:** gender equity, nursing, journal article, journal impact factor, cross-sectional studies

## Abstract

**Background:**

Scientific activity has been connected to the proven inequality between women and men. To examine the state of gender equality in nursing research by analyzing the representation of male and female as editors and as authors of articles published in scientific journals.

**Method:**

A cross-sectional study was carried out between September-2019 and May-2020. All the scientific publications published in 115 nursing journals indexed in the Journal Citation Reports in the years 2008, 2013, and 2017 were chosen as analysis units. The main variables studied were gender of the “journal editor”; gender of the “first author”, “last author”, “corresponding author”, and “first author in funded articles”. Descriptive and inferential analysis was performed.

**Results:**

The proportion of male editors in 2008, 2013, and 2017 was 23.3, 19, and 18.5% respectively, with a male/female ratio of 1:3, 1:4 and 1:5. Male editors are mainly found in the journals of the first quartile (Q1 = 33.8%, ratio1:2), compared to the journals of the fourth quartile (Q4 = 6.6%, ratio1:14), *p* < 0.01. The male authorship position was “last author” (30.9%, ratio1:2), “corresponding author” (23.3%, ratio 1:3), “first author” (22.1%, ratio 1:4) and “first author in funded articles” (21.8%, ratio 1:4). Furthermore, in 19.5%, of the articles there were more male authors. The percentage of articles with male authorship increased from 2008 to 2017, “first author” (21.1–23.4%; *p* < 0.01), “last author” (30.0–31.1%; *p* = 0.22), “corresponding author” (22.5–24.2; p = 0.01), and “first author in funded articles” (18.1–25.9%; *p* < 0.001).

**Conclusions:**

Men are over-represented in the editor role in the most prestigious nursing journals. There are a higher proportion of male authors in the main positions of authorship.

## Introduction

Equality between women and men is a universally recognized, fundamental legal principle (1). However, gender inequality is a worldwide reality and, despite being a historical objective, its elimination is far from being achieved (2). This inequality is based on false sexist prejudices that create different social expectations about men and women's behavior and achievements (3). The sexual division of productive and reproductive work has consolidated a hierarchy of power and privileges, reinforcing a systemic inequality and restricting women's opportunities (3).

Even in the countries with the greatest progress in gender equality, women continue to be underrepresented in the most influential and best-paid jobs, and within the same work activity, they reach, to a lesser extent, the most privileged positions. Gender parity is a necessary requirement for equality. The balanced participation of men and women in positions of power and decision-making favors that the interests, needs, and merits of both are equally valued (4).

Scientific activity has been shown to be connected to the proven inequality of women in the workplace (5). Although women have reached the highest academic levels and have achieved an important presence in academic and research fields, few of them occupy leadership positions or achieve recognition from the scientific community (6).

Both participation in the editorial board of scientific journals and the chief editor position, which is the journal's main editorial position, is chosen from among people of recognized scientific prestige and are indicators of leadership in an academic field. The editor assumes the responsibility of maintaining the journal's scientific quality and has a decisive influence on the dissemination of knowledge of the discipline. Even in the biosanitary field, where women are the majority, they continue to be underrepresented on the journals' scientific committees, and even more so at the head of the editorial staff (7).

The number of articles published in prestigious journals is another indicator of researchers' power of influence in their field of knowledge. Scientific publications are the main means of disseminating scientific knowledge and are the main measure of the researchers' productivity (8). Authorship confers credit to the researcher and has academic, social, and financial importance. The number of publications and grants substantially improves the researcher's economic gain, possibilities of promotion, and power within their research field, thus influencing women scientists' career perspectives and visibility (9). Studying authorship of scientific publications from a gender perspective is a proven way to measure gender bias within the scientific community. Analysis of authorship of publications in high-impact journals determines the quality of women's representation in a given discipline. Likewise, authors' position in an article is relevant to evaluate the responsibility in a project: first authorship, last authorship, and correspondence author are considered of greater prestige (10).

Gender bias in the authorship of publications and the leadership of editorial boards has been analyzed in many disciplines, mainly in those traditionally masculinized, where the integration of women is a minority or relatively recent (10–12). Traditionally feminized professions (female workers equal to or >65%) such as nursing do not seem to show the same scientific production as those predominantly masculinized (male workers equal to or >65%). Nevertheless, in recent years, nursing contribution to biomedical research has increased substantially due to higher professional development (13–15).

The presence of men in the nursing profession is a minority. The “State of the World's Nursing Report 2020” estimates that only 10% of the nursing staff are men. This minority of male nurses occupy different positions than women and tend to work in emergency care, critical care, or mental health units, while they are less likely to work in maternal and pediatric units. They more frequently occupy positions related to management and teaching, and positions of greater responsibility and leadership that provide them with greater social visibility and higher levels of remuneration. This greater career advancement of men in women-dominated professions was designated by William in 1992 as a 'glass escalator' to illustrate that men can achieve promotion or advancement faster than their women colleagues, thus establishing gender differences in the profession (16).

Several studies have documented the gender gap in nursing by analyzing wage inequality (16–18). However, few studies measure male and female nurses' presence as the editor and author of scientific publications. We hypothesized that male nurses are over-represented in nursing research and occupy the most privileged positions. This reality needs to be documented since the figures help to identify the dimensions and evolution of inequalities, while at the same time, they can serve to dismantle false beliefs about equality that justify inaction.

This study aimed to examine the state of gender equality in nursing research by analyzing male and female nurses' representation as editors of scientific journals indexed in Journal Citation Reports (JCR) and as authors of articles published in these journals over a decade (from 2008 to 2017).

We addressed the following research questions in this study: (1). Does the representation of male and female nurses in the roles of editor and author match nurses' representation in the nursing profession? (2). Has the representation of male and female nurses in editor and author roles changed over the decade 2008–2017? (3). Is there an association between the gender of the journal editors and the journal quartile (Q)? (4). Is there a relationship between the gender of the journal editor and the greater or lesser representation of men and women in the authorship of articles published in the journals?

## Methodology

### Design

A cross-sectional study was carried out between September 2019 and May 2020.

### Sample

All the scientific publications published in Journal Citation Reports (category: nursing) in the years 2008, 2013, and 2017 were chosen as units of analysis.

### Selection criteria

Those articles in which the gender of the authors could not be determined, either because the meaning of the initials of the name was not identified, or because of difficulties in knowing the gender of Asian authors, were excluded from the analysis. Articles with multiple authorship in which there was a percentage of equality (50%) between men and women were also excluded from the analysis of the variable “majority of men or women”. We have added the comment: In these publications with equal numbers of male and female authors, it was considered that there was no inequality.

### Procedure

All original articles and reviews published in Journal Citation Reports (category: nursing) in 2008, 2013, and 2017 were reviewed.

A group of researchers was trained to collect the data during a 2-h training session to access, evaluate, and record each journal's data. Sets of two researchers independently assessed the data. Results from these independent assessments were compared, and in case of disagreement, the researchers discussed their ratings and established consensus. A third researcher was consulted when discrepancies arose. Author gender was determined using the online database http://genderize.io. This database includes >200,000 names and determines each name's probability of being male or female given the distribution for these names in the database. When individuals' names were not listed in genderize.io, or ad less than a 95% probability of being one gender, an Internet search was used to determine gender. In this regard, individual web pages or entries in online databases, including photographs of the individual or other information suggesting their gender, were performed. We were able to genderize ~98% of all subjects.

### Variables

The following variables were studied: gender of the journal editor; gender of the first author; gender of the last author; gender of the corresponding author; dominant gender of the authors; position of the journal; existence of funding. Single-authored articles were counted as first author only.

Only “men” and “women” options were considered when assessing gender identity regarding the position of the journal, distribution by Quartile (from Q1 to Q4) was evaluated, when abstracts included funding, and the gender of the first author of those articles was assessed.

### Data analysis

The Statistical Package for Social Sciences (SPSS 25, SPSS Inc., Chicago, IL, USA) software was used for the statistical analysis. For the descriptive analysis, data were expressed as frequencies, percentages, and men/women ratio. For the inferential analysis, to measure the association between gender of editors and authors, between gender of authors and editors with years of publication, and between gender of authors and editors with position/Qs, Chi-square test or Fisher exact test for independent samples were performed. The McNemar test was used to assess changes in the gender of the editor.

### Ethical considerations

The study did not require any ethics committee authorization because no humans or living beings were involved, nor any data accessed that might compromise privacy or intimacy. The researchers do not declare any ethical or moral conflict, nor have they received any funding or benefits from industry or elsewhere to conduct this study.

## Results

The articles of 115 (99.13%) journals were analyzed, out of a total of 116. The gender “man” vs. “woman” was attributed for 103, 105 and 108 editors in the years 2008, 2013, and 2017 respectively. The first author of 23,001 articles, the last author of 17,635 articles, and the corresponding author of 21,694 articles was determined. A total of 1,293 articles included the existence of funding. The 85% of the journals were published in the United States (65%) and the United Kingdom (15%).

### Editors

**Figure 1:**
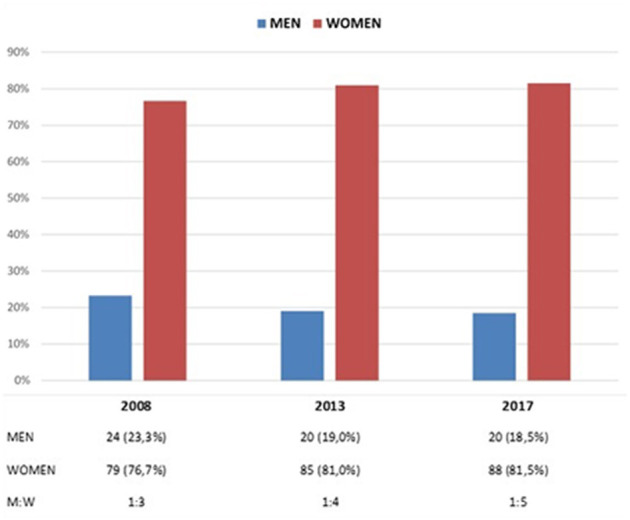
Men/women editors in 2008, 2013, and 2017 years.

In 2008, 23.3% of the nursing journals analyzed had a male editor; 10 years later, the percentage drops to 18.5% (Figure 1). This drop of 4.8% in male representation was not significant (McNemar test, *p* = 0.125). The decline was more evident between 2008 and 2013 (4.3%), and then the decrease slowed down between 2013 and 2017 (0.5%). In 2008, there was a ratio of 1 male editor for every 3 female editors, 1:4 in 2013 and 1:5 in 2017 (Figure 1).

**Figure 2:**
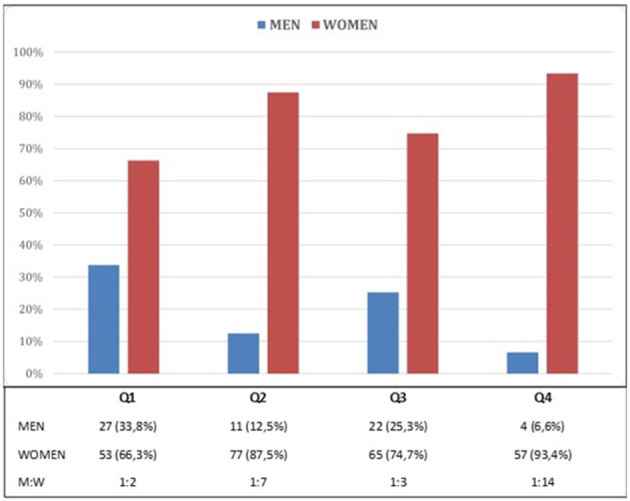
Men/women ratio by quartile.

Among the journals in the first quartile (Q1), the position of editor is held by one man for every two women (33.8% vs. 66.3%); in the fourth quartile journals (Q4), the ratio drops to 1 man for every 14 women (6.6% vs. 93.4%) (Figure 2). There were significant differences in the proportion of men and women editors according to the journal Q (test χ^2^ = 20.74, 3gl, *p* < 0.01).

**Figure 3:**
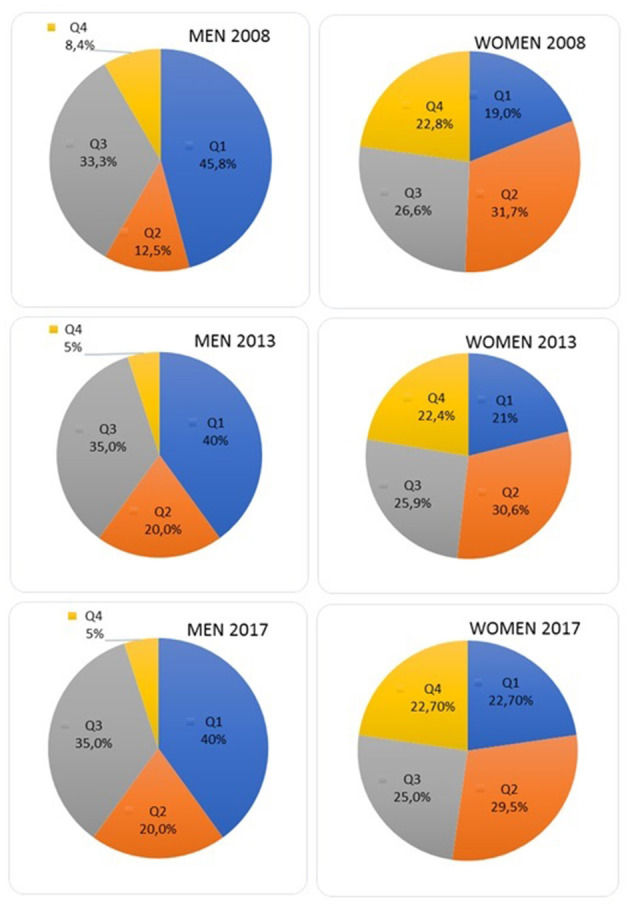
Ratio of men/women by year and quartile.

The distribution of journal editors in each quartile, according to gender and year, is shown in Figure 3. Male editors' presence was high among Q1 journals (45.8% in 2008 vs., 40% in 2013 and 2017) and low among Q4 journals (8.4% in 2008 vs., 5% in 2013 and 2017). If we group the first two quartiles (Q1 and Q2) and the last two quartiles (Q3 and Q4), the editors' distribution in these ten years was neither significant nor in men (χ^2^ = 0.01, 1gl, *p* = 0.91) or in women (χ^2^ = 0.04, 1gl, *p* = 0.83).

### Authors

In the total number of articles reviewed, the position of authorship where men are best represented is in the last position (one man for every two women), followed by the position of corresponding author (one man for every three women) and finally the position of first author and first author in funded articles (one man for every four women). Table 1 details the percentage of articles with male/female authorship by publication year and quartile, at all authorship positions.

**Table 1:** Descriptive results of study: first author, last author, corresponding author, most male authors, and first author in funded articles.

	**First author (***N*** = 23,001)**			**Last author (***N*** = 17,635)**			**Corresponding author (***N*** = 21,694)**			**Most male authors (***N*** = 20,168)**			**First author in funded articles (***N*** = 1,293)**		
	**M**	**W**	**M:W**	**M**	**W**	**M:W**	**M**	**W**	**M:W**	**M**	**W**	**M:W**	**M**	**W**	**M:W**
**Year**	***n*** **(%)**	***n*** **(%)**		***n*** **(%)**	***n*** **(%)**		***n*** **(%)**	***n*** **(%)**		***n*** **(%)**	***n*** **(%)**		***n*** **(%)**	***n*** **(%)**	
2008	1,484 (21.1)	5,548 (78.9)	1:4	1,434 (30.0)	3,341 (70.0)	1:2	1,454 (22.5)	5,010 (77.5)	1:3	1,169 (19.3)	4,881 (80.7)	1:4	59 (18.1)	266 (81.9)	1:5
2013	1,627 (21.5)	5,944 (78.5)	1:4	1,885 (31.5)	4,104 (68.5)	1:2	1,642 (22.9)	5,532 (77.1)	1:3	1,290 (19.5)	5,321 (80.5)	1:4	88 (19.7)	358 (80.3)	1:4
2017	1,966 (23.4)	6,432 (76.6)	1:3	2,136 (31.1)	4,736 (68.9)	1:2	1,951 (24.2)	6,105 (75.8)	1:3	1,484 (19.8)	6,023 (80.2)	1:4	135 (25.9)	387 (74.1)	1:3
Total	5,077 (22.1)	17,924 (77.9)	1:4	5,455 (30.9)	12,181 (69.1)	1:2	5,047 (23.3)	16,647 (76.7)	1:3	3,943 (19.5)	16,225 (80.5)	1:4	282 (21.8)	1,011 (78.2)	1:4
**Quartile**															
Q1	598 (21.8)	2,148 (78.2)	1:4	785 (33.5)	1,556 (66.5)	1:2	623 (22.7)	2,120 (77.3)	1:3	527 (20.6)	2,029 (79.4)	1:4	50 (30.3)	115 (69.7)	1:2
Q2	447 (21.2)	1,664 (78.8)	1:4	521 (28.3)	1,323 (71.7)	1:3	436 (21.9)	1,551 (78.1)	1:4	370 (19.3)	1,550 (80.7)	1:4	19 (23.2)	63 (76.8)	1:3
Q3	604 (32.0)	1,285 (68.0)	1:2	565 (36.8)	971 (63.2)	1:2	612 (34.5)	1,272 (67.5)	1:2	389 (26.6)	1,075 (73.4)	1:3	30 (27.3)	80 (72.7)	1:3
Q4	317 (19.1)	1,341 (80.9)	1:4	265 (23.2)	876 (76.8)	1:3	280 (19.4)	1,162 (80.6)	1:4	198 (12.6)	1,369 (87.4)	1:7	35 (21.8)	129 (78.2)	1:4

Q1, Quartile 1; Q2, Quartile 2; Q3, Quartile 3; Q4, Quartile 4; %, percentage; N / n, frequency; M, men; W, women; M:W, ratio male/female.

The percentage of articles with male authorship increased from 2008 to 2017: 2.3% first authorship (21.1 vs. 23.4%; *p* < 0.01), 1.1% last authorship (30 vs. 31.1%; *p* = 0.22), 1.7% correspondence authorship (22.5 vs. 24.2; *p* = 0.01), 0.5% of articles with most male authors (19.3 vs. 19.8%; *p* = 0.52) and 7.8% in the position of first author of funded articles (18.1 vs. 25.9%; *p* < 0.001) (Table 1).

Regarding male representation as authors of articles published in journals with impact factor, in all authorship positions, men are better represented in Q1 journals than in Q4. This higher percentage of representation of men in the journals with more impact is statistically significant in all the observed assumptions: first author (Q1 = 21.8 vs. Q4 = 19.1%; *p* = 0.03), last author (Q1 = 33.5 vs. Q4 = 23.2%; *p* < 0.01), corresponding author (Q1 = 22.7% vs. Q4 = 19.4%; *p* = 0.01), articles with more male authors (Q1 = 20.6% vs. Q2 = 12.6%; *p* < 0.01), first author of funded articles (Q1 = 33.3% vs. Q4 = 21.8%; *p* = 0.04). In the latter case, the ratio of man/women increases from one man for every two women in the Q1 to one man for every four women in the Q4 (Table 1).

### Relationship between the gender of the editor and the gender of the authors

Male authors' representation has been greater in the journals with a male editor than in those with a female editor. Table 2 shows the statistically significant association between the gender of the journal editor and the gender of the first author, last author, and correspondence author (*p* < 0.01). However, although the percentage of articles funded with male first author compared to female authors is higher when the editor is male, the association was not statistically significant (*p* = 0.143).

**Table 2:** Relations gender of authors/gender of editors.

**Gender of authors**	**Gender of editors**		**Chi-square test**
	**Men**	**Women**	
	***n*** **(%)**	***n*** **(%)**	***p***
**First author**			
Mane	1,143 (24.3)	3,552 (75.7)	< 0.01
Woman	3,144 (19.2)	13,251 (80.8)	
**Last author**			
Man	1,372 (26.8)	3,745 (73.2)	< 0.01
Woman	2,270 (20.1)	8,998 (79.8)	
**Corresponding author**			
Mane	1,170 (24.3)	3,637 (75.7)	< 0.01
Woman	3,042 (19.5)	12,533 (80.5)	
**Most authors**			
Man	930 (25.4)	2,733 (74.6)	< 0.01
Woman	2,679 (18.3)	12,000 (81.7)	
**First author in funded articles**			
Man	50 (19.5)	207 (80.5)	0.143
Woman	147 (15.6)	793 (84.4)	

%, percentage; n, frequency.

## Discussion

In this study, we examined gender parity in men and women's distribution as the editor of prestigious scientific journals in nursing and as the author. We found no parity between male and female nurses, which confirms our hypothesis that male nurses disproportionately occupy promising research career positions. Male editors are mainly grouped in the most prestigious journals, and there is a greater presence of men in both the last author positions and in the authorship of funded articles. In addition, women are more likely to be the first authors, but less likely to be the last authors.

The representation of women in authorship has worsened over the years studied, most notably in funded articles' authorship. An interesting association was found between the gender of the editor and that of the authors.

### Parity between male editors and female editors

This study shows that, in the role of editor, female nurses suffer a double inequality. On the one hand, there is a horizontal inequality: the representation of male nurses in the role of editor is two to three times greater than would be the case if the number of nursing editors was proportional to the number of nurses in the profession. On the other hand, there is a vertical inequality: male editors are mostly grouped in Q1 journals. The percentage of male editors with respect to female editors decreased over the years, although not significantly, and the concentration of editors in the most prestigious journals persists. This gender inequity among editors is documented in other studies (19, 20).

The lack of parity in the editor's role is significant since editors are the main guarantors of the nursing literature quality (21, 22). The editor appoints peer reviewers who judge the manuscript's quality and make recommendations for improvement, but the ultimate decision of whether to publish the article is made by the editor (23). The gender of the editor and reviewers should be irrelevant to the acceptance or otherwise of the manuscript. Nevertheless, our data support a homophilic relationship in the publication process in nursing journals, since men publish more in journals with male editors, and vice versa. This relationship is maintained in the 3 years observed and in all types of authorship, except in the authorship of funded articles. This homophilic relationship is documented in the different scientific knowledge fields (20, 24). A recent study on the review and acceptance process of articles in biosciences between 2012 and 2017 showed that this relationship becomes more noticeable when men occupy the last author position (24).

It is plausible that, as has been observed in other disciplines (19, 20, 25, 26), there is a gender bias in the relationship between editors and nurse reviewers. Male editors invite fewer women than men to be reviewers (20, 25), while female editors invite more women to review studies than male editors (19). Women are less likely than men to be invited to review articles, and women more often decline to be reviewers because of lack of time, overwork, or not considering themselves experts in the field (25). A study that analyzed gender parity in the invitation of writing comments in 2,459 medical journals by their editors found that the number of invitations to women was 21% less than men with similar research experience, number of publications, and impact of citations (27). The impartiality of editors and reviewers in the selection process of studies to be published has been questioned in several studies, and the results are contradictory according to the scientific area explored (28–30).

On the other hand, it is well known that belonging to a minority (a man in a feminized profession) can favor the professional promotion of other members of that minority (31). If we apply this principle to nursing research, it would explain that if men nursing editors perceive male authors and themselves as part of a minority group within the profession, this could condition their decision to accept their manuscripts more readily, facilitating their passage into the review process.

### Parity of men and women in articles authorship

The over-representation of men in articles authorship in the most prestigious nursing journals corroborates the data on discrimination against women in the authorship of scientific literature in other disciplines (6, 9, 11, 12, 19, 27, 32). According to our data, men's over-representation did not decrease between 2008 and 2017, although it increased in all authorship positions. This increase in male authorship is not consistent with authorship studies in other traditionally male scientific areas, where female authorship is growing at the expense of male authorship (10, 12, 19), although this increase is small. According to the study of Holman et al. (9), it will take decades for women scientists to reach parity with their men counterparts.

The increase in male authorship in our study does not seem to respond to an increase in men's proportion in the nursing profession. According to data provided by the Center for Interdisciplinary Health Workforce Studies in 2017, the percentage of men nurses in the USA, the country where the largest number of nursing journals reviewed in this study are published, remained constant between 2013 and 2017, at around 11%. However, the number of women registered nurses who earned either masters or doctoral degrees between 2003 and 2015 increased nearly five-fold, from 7,600 to 37,000, while the increase among men was smaller, from 1,000 to 4,000. In the UK, the proportion of men was 10.5% in 2013 and 10.7% in 2018 (33). Authors from the USA and the UK accounted for 40% of all publications between 2012 and 2017 in the six nursing journals with the highest impact factor (34).

The greater representation of men in the last author position and women in the first author position coincides with another study's results (19). The last author position is where senior researchers, i.e., those with longer research careers or expertise, are most often found (35). It is among the most productive authors that the gender gap widens (6, 27), probably because women are more likely to drop out of the scientific career than men (36–40). First female authors, who are presumably at the beginning of their careers and who carry a greater weight of research, should progress over the years and assume leadership positions in research and therefore more often occupy the last author position (41). Nevertheless, while in other studies the over-representation of men in the last author position decreases over time (19) in our study it remains, which makes us think that the causes of abandonment of the research career among women nurses persist.

With respect to the corresponding author position in the 3 years studied, we observe that the proportion of women is always somewhat lower than the percentage of first authors. It is considered that corresponding authors are usually the highest ranking researchers among those who generated the idea of the research (37). This difference between the percentage of first female authors and corresponding female authors has also been found in other studies (12, 19).

On the other hand, the lower representation of women in the first author position of funded articles found in this study and the trend toward a lower representation of women in this type of authorship in the decade analyzed is very worrying in the nursing field. This inequality in funding has been shown in other research in the biomedical area (42–44). This discrimination of women in the granting of funding for their studies would respond to the “Matilda effect” (Mattew effect), a term contributed in 1993 by Margaret Rossiter to illustrate those situations in which the efforts and scientific achievements of women do not receive the same recognition as those of men (45).

In our study, the highest presence of female authors was concentrated in the journal with the lowest impact factor (Q4), which is consistent with studies from other disciplines: mathematics (46), biology (47), or biomedicine (48), although in other studies conducted in computational biology (47) or biomedical professions (41) this difference does not exist.

### Gender analysis of the unequal representation of men and women in nursing research

Gender disparity in science affects all disciplines and all countries worldwide (6), but there are aspects of the nursing profession that we need to reflect upon. The data observed in this study cannot be analyzed without considering the intricate influences of gender on the process of nursing professionalization and the deep-rooted ideologies and power relations that permeate nursing practice (49, 50).

According to Cottingham (51), this over-representation of male nurses in the research career was already documented three decades earlier by Ryan and Portes. Despite being a minority in the USA and UK, the authors showed that male nurses had a disproportionately large nursing research presence. At that time, in the UK, 8.8% of men in the profession contributed to 40% of the publications in the three journals they analyzed, and in the USA, the percentage of male nursing professors (6.5%) was double that of men nurses (3.1%). The authors of this study were criticized by some male nurses who did not accept that there were benefits, but rather the opposite: they felt that they were part of a male minority discriminated against within a female-dominated profession.

The construction of masculinity within a profession that is strongly linked to the “feminine” gender can be conflictive for some male nurses (52). One way to preserve their masculinity is to differentiate their tasks within the profession itself (53), preferring highly technical, medicalized care, or leadership positions more in line with traditional masculinity values.

The academic and research career provides men nurses with the possibility to re-label their profession (54, 55) and avoid conflict. Some authors believe that a greater number of men in the nursing profession would help break the stereotypes related to the nursing profession and raise the profession's social prestige (52, 55, 56). However, we consider that, based on the known data, it is unlikely that an increase in the number of men would improve the parity data of women nurses in the scientific career, in the same way, that the increase of women in other scientific areas has not significantly decreased the gender gap within these disciplines, especially when the most prestigious positions are analyzed (11, 39). Recent research comparing the inequality between men and women in scientific careers between countries and disciplines finds that, paradoxically, the increase in the number of women in the last 60 years in mainly men scientific areas has been accompanied by an increase in the gender gap, both in the scientific productivity and in the impact of their publications (6). The problem does not seem to be the number of nurses, but rather the prejudices that favor the rise of men (57) and burden women's academic and research careers (58). Berkery's study (57) analyzed the perception of male and female nurses about the characteristics that define nurse managers and their association with gender stereotypes, and found that while women did not associate stereotypes of masculinity and femininity with the role of manager, director, or manager, male nurses typified the managerial role in favor of men.

Female nurse researchers are likely to face the same obstacles and difficulties as other women scientists. Several studies have revealed that women's scientific production is conditioned by an implicit bias or unconscious prejudice, according to which people act based on thought patterns or ideas that often lead them to engage in discriminatory behavior of which they are not aware (58–61). Analysis of discrimination against women in the research career in light of this concept has helped to reveal a widespread gender bias among academic and research staff themselves, leading to the belief that men have greater research skills and competencies and greater leadership potential, which tend to favor them (62). Hence, women are less likely to be cited in the scientific literature, to obtain leadership positions, to earn better salaries, or to receive funding for their projects, and are more likely to leave the research career prematurely (3, 9, 63, 64).

The discrepancy between the stereotypes of women and researchers may be even more profound in women nurses than in women physicists, physicians, or engineers (3, 52). While parity appears to be improving among medical professionals (10, 41), it does not appear to be so among nurses, according to data from this study. A combination of social, educational, and occupational factors discourages female nurses from entering leadership positions or causes increased attrition in professional development that favors their abandonment (57). This discrimination in the scientific nursing field may be one more aspect that would explain part of the proven salary discrimination of female nurses (16–18, 65), which is greater than that observed in other professions with a majority of women and which seems not to have decreased in recent years (16).

### Limitations of the study

One of the difficulties of this study was identifying the proportion of men and women in the nursing profession with a university education. Data on the workforce of nursing professionals are very diverse across countries and includes different professional categories with and without university degrees (50). We have accepted that there are around 10% of men in the nursing profession with university training (WHO) because it is consistent with the percentages of male nurses registered in the USA (11.1%), Europe (10%), Canada (9%), or Australia (11.7%). Those are the main countries in which the journals are published and contribute the greatest number of authors. In addition, data from some countries include midwives among nursing professionals, but in others, they count midwives independently. Considering that the minority of men among midwives is even more pronounced than in nursing and that this group is included among the editors and authors of the journals reviewed, we consider that the percentage of 10% of men is not overestimated.

Other important limitations have been the impossibility of determining the gender of the author when identified in the manuscript with an initial capital letter; when this was not possible, attempts were made to identify it through other means, such as meta-searches, research platforms, or public profiles. We also found it difficult to identify authors/editors in Asian languages, which led to the exclusion of one journal and low identification in another, to simplify analysis. Also, the difficulty of identifying the gender of authors within the same article is responsible for the differences observed in the number of first authors identified, last authors, or corresponding authors. In the latter case, moreover, we found it difficult to detect authorship if it was not explicitly visible.

In the other hand, the affiliations of the included articles were not consulted for this study. Although we believe that it will not be very influential in the overall results, it is possible that some articles published in these nursing journals have been published by professionals from other health sciences (such as psychologists or physiotherapists, for example). Therefore, in future studies this should be a bias to be taken into account.

Although we have explored the gender approach in funded journals, we have only analyzed publications where funding was stated in the summary of the article, not accessing the full articles.

We have made an effort to explain our data and argue about the possible causes that generate them. However, this study's data only shows the gender gap in nursing but cannot determine its causes.

### Practical application

There is a lack of data on the gender gap in the nursing research career and an in-depth analysis of the factors that influence it. There is a need to document and recognize the biases against female nurse scientists to reduce them in each country's context. We do not believe that the debate is how to incorporate more men into the nursing profession, but rather how to empower women nurses in the research career and break down stereotypes associated with women and nurses that burden their careers. This publication opens an important line of research on the existence of a “glass escalator” for men in nursing research and a “glass ceiling” for women. And that work and research must be done to minimize these phenomena.

## Conclusions

Male nurses are over-represented in the role of editor and author of articles in prestigious nursing journals and there is a greater proportion of male authors who publish in journals that have male editors.

Female editors are more often concentrated in journals with a lower level of impact, and female authors occupy authorship positions more related to the initial phases of the research career and less to a long and productive academic and research career, which suggests that there is a slowdown in women's research careers or an abandonment of them. This would confirm the existence of a “glass escalator” for men in nursing research.

## Data availability statement

The raw data supporting the conclusions of this article will be made available by the authors, without undue reservation.

## Author contributions

VG-C, CS-O, LC-B, and RJ-V designed the study. RR, CS-O, IS-A, EM-S, and RJ-V analyzed and interpreted the data. VG-C, RR, CS-O, LC-B, IS-A, EM-S, and RJ-V wrote the work and critically reviewed it. All authors read and approved the final manuscript.

## References

[1] United Nations. Convention on the Elimination of All Forms of Discrimination against Women. (1979). Available online at: http://www.un.org/womenwatch/daw/cedaw/12289228

[2] World Economic Forum. Global Gender Gap Report 2020. (2019). Available online at: http://www3.weforum.org/docs/WEF_GGGR_2020.pdf

[3] Heise L Greene ME Opper N Stavropoulou M Harper C Nascimento M. Gender inequality and restrictive gender norms: framing the challenges to health. Lancet. (2019) 393:2440–54. 10.1016/S0140-6736(19)30652-X 31155275

[4] Council of Europe. (2003). Recommendation Rec (2003). Balanced Participation of Women and Men in Political and Public Decision Making. Strasbourg: Council of Europe.

[5] Hoa NT Thuong NTT Clapham HE Thu TTA Kestelyn E Thwaites CL. Increasing women's leadership in science in Ho Chi Minh City. Lancet. (2019) 393:523–4. 10.1016/S0140-6736(18)32090-7 30739681

[6] Huang J Gates AJ Sinatra R Barabási AL. Historical comparison of gender inequality in scientific careers across countries and disciplines. Proc Natl Acad Sci U S A. (2020) 117:4609–16. 10.1073/pnas.1914221117 32071248 PMC7060730

[7] Sarna KV Griffin T Tarlov E Gerber BS Gabay MP Suda KJ. Trends in gender composition on editorial boards in leading medicine, nursing, and pharmacy journals. J Am Pharm Assoc. (2020) 60:1–6. 10.1016/j.japh.2019.12.018 31953121

[8] Ioannidis JPA. How to make more published research true. PLoS Med. (2014) 11:e1001747. 10.1371/journal.pmed.1001747 25334033 PMC4204808

[9] Holman L Stuart-Fox D Hauser CE. The gender gap in science: How long until women are equally represented? PLoS Biol. (2018) 16:1–20. 10.1371/journal.pbio.2004956 29672508 PMC5908072

[10] Brinker AR Liao JL Kraus KR Young J Sandelski M Mikesell C. Bibliometric analysis of gender authorship trends and collaboration dynamics over 30 years of spine 1985 to 2015. Spine. (2018) 43:E849–54. 10.1097/BRS.0000000000002562 29438219

[11] Aguinis H Ji YH Joo H. Gender productivity gap among star performers in STEM and other scientific fields. J Appl Psychol. (2018) 103:1283–306. 10.1037/apl0000331 30024197

[12] Dynako J Owens GW Loder RT Frimpong T Gerena RG Hasnain F. Bibliometric and authorship trends over a 30 year publication history in two representative US sports medicine journals. Heliyon. (2020) 6:e03698. 10.1016/j.heliyon.2020.e03698 32258505 PMC7114749

[13] Aarts C. Strategy for nursing research in Sweden. Investig y Educ en Enferm. (2017) 35:5–7. 10.17533/udea.iee.v35n1a01 29767918

[14] Boulton MG Beer S. Factors affecting recruitment and retention of nurses who deliver clinical research: a qualitative study. Nurs Open. (2018) 5:555–66. 10.1002/nop2.167 30338101 PMC6177552

[15] Cole A. Why nursing research matters. Nurs Stand. (2016) 30:16–8. 10.7748/ns.30.32.16.s20 27049991

[16] Wilson BL Butler MJ Butler RJ Johnson WG. Nursing gender pay differentials in the New Millennium. J Nurs Scholarsh. (2018) 50:102–8. 10.1111/jnu.12356 29116683

[17] Muench U Dietrich H. The male-female earnings gap for nurses in Germany: a pooled cross- sectional study of the years 2006 and 2012. Int J Nurs Stud. (2019) 89:125–31. 10.1016/j.ijnurstu.2017.07.006 28716298

[18] Muench U Sindelar J Busch SHBP. Salary differences between male and female registered nurses in the United States. JAMA. (2015) 313:1265–7. 10.1001/jama.2015.1487 25803350

[19] Fox CW Paine CET. Gender differences in peer review outcomes and manuscript impact at six journals of ecology and evolution. Ecol Evol. (2019) 9:3599–619. 10.1002/ece3.4993 30962913 PMC6434606

[20] Helmer M Schottdorf M Neef A Battaglia D. Gender bias in scholarly peer review. eLife. (2017) 6: e21718. 10.7554/eLife.21718.012 28322725 PMC5360442

[21] Freda MC Kearney MH. Ethical issues faced by nursing editors. West J Nurs Res. (2005) 27:487–99. 10.1177/0193945905274906 15870245

[22] Odom-Forren J. Editorial independence and the society editor. Nurse Author Editor. (2017) 27:1–9. 10.1111/j.1750-4910.2017.tb00249.x

[23] Watson R. Peer review and scholarly publishing. Nurse Author Editor. (2020) 30:1–5. 10.1111/j.1750-4910.2020.tb00055.x

[24] Murray D Siler K Bonobo TS Chan WM Collings AM Raymond J. (2018). Gender and international diversity improves equity in peer review. BioRxiv. 400515. 10.1101/40051532633721

[25] Lerback JHB. Journals invite too few women to referee. Nature. (2017) 541:455–7. 10.1038/541455a 28128272

[26] Mauleón E Hillán L Moreno L Gómez I Bordons M. Assessing gender balance among journal authors and editorial board members. Scientometrics. (2013) 95:87–114. 10.1007/s11192-012-0824-4

[27] Thomas EG Jayabalasingham B Collins T Geertzen J Bui C Dominici F. Gender disparities in invited commentary authorship in 2459 medical journals. JAMA Network Open. (2019) 2:e1913682. 10.1001/jamanetworkopen.2019.13682 31642926 PMC6820037

[28] Cox AR Montgomerie R. The cases for and against double-blind reviews. PeerJ. (2019) 7:e6702. 10.7717/peerj.6702 30972261 PMC6450368

[29] Edwards HA Schroeder J Dugdale HL. Gender differences in authorships are not associated with publication bias in an evolutionary journal. PLoS ONE. (2018) 13:e0201725. 10.1371/journal.pone.0201725 30157231 PMC6114708

[30] Fox CW Burns CS Muncy AD Meyer JA. Gender differences in patterns of authorship do not affect peer review outcomes at an ecology journal. Funct Ecol. (2016) 30:126–39. 10.1111/1365-2435.12587

[31] Manlove KR Belou RM. Authors and editors assort on gender and geography in high-rank ecological publications. PLoS ONE. (2018) 13:1–13. 10.1371/journal.pone.0192481 29420647 PMC5805316

[32] West JD Jacquet J King MM Correll SJ Bergstrom CT. The role of gender in scholarly authorship. PLoS ONE. (2013) 8. 10.1371/journal.pone.0066212 23894278 PMC3718784

[33] The UK Nursing Labour Market Review. (2018). Available online at: www.rcn.org.uk

[34] Giménez-Espert M del C Prado-Gascó VJ. Bibliometric analysis of six nursing journals from the Web of Science, 2012–2017. J Adv Nurs. (2019) 75:543–54. 10.1111/jan.13868 30289557

[35] Duffy MA. Last and corresponding authorship practices in ecology. Ecol Evol. (2017) 7:8876–87. 10.1002/ece3.3435 29152184 PMC5677469

[36] Adamo SA. Attrition of women in the biological sciences: workload, motherhood, and other explanations revisited. Prof Biol. (2013) 63:43–48. 10.1525/bio.2013.63.1.9

[37] Macaluso B Larivière V Sugimoto T Sugimoto CR. Is science built on the shoulders of women? A study of gender differences in contributorship. Acad Med. (2016) 91:1136–42. 10.1097/ACM.0000000000001261 27276004

[38] Shen YA Webster JM Shoda Y Fine I. Persistent underrepresentation of women's science in high profile journals. bioRxiv [Preprint]. (2018). Available online at: https://www.biorxiv.org/content/biorxiv/early/2018/03/08/275362.full.pdf

[39] Shen H. Mind the gender gap. Nature. (2013) 495:22–4. 10.1038/495022a 23467149

[40] Stewart A Valian V. An inclusive academy: achieving diversity and excellence. MIT Press. (2018). 10.7551/mitpress/9766.001.0001

[41] Giner-Soriano M López-Pereiro O Zabaleta-del-Olmo E Pons-Vigués M Morros R Gómez-Lumbreras A. Bibliometric analysis of female authorship in original articles in the journal ATENCIÓN PRIMARIA. Atencion Primaria. (2019) 53:12–8. 10.1016/j.aprim.2019.11.002 31898990 PMC7752960

[42] Burns KEA Straus SE Liu K Rizvi L Guyatt G. Gender differences in grant and personnel award funding rates at the Canadian Institutes of Health Research based on research content area: a retrospective analysis. PLoS Med. (2019) 16. 10.1371/journal.pmed.1002935 31613898 PMC6793847

[43] Crowley MJ Al-Khatib SM Wang TY Khazanie P Kressin NR Krumholz HM. How well does early-career investigators' cardiovascular outcomes research training align with funded outcomes research? Am Heart J. (2018) 196:163–9. 10.1016/j.ahj.2017.09.008 29421009

[44] García-Calvente M del M Ruiz-Cantero MT del Río-Lozano M Borrell C López-Sancho MP. Desigualdades de género en la investigación en salud pública y epidemiología en España (2007-2014). Gaceta Sanitaria. (2015) 29:404–11. 10.1016/j.gaceta.2015.07.013 26404162

[45] Lincoln AE Pincus S Koster JB Leboy PS. The matilda effect in science: awards and prizes in the US, 1990s and 2000s. Soc Stud Sci. (2012) 42:307–20. 10.1177/0306312711435830 22849001

[46] Mihaljević-Brandt H Santamaría L Tullney M. The effect of gender in the publication patterns in mathematics. PLoS ONE. (2016) 11:e0165367. 10.1371/journal.pone.0165367 27780266 PMC5079651

[47] Bonham KS Stefan MI. Women are underrepresented in computational biology: An analysis of the scholarly literature in biology, computer science and computational biology. PLoS Comput Biol. (2017) 13:e1005134. 10.1371/journal.pcbi.1005134 29023441 PMC5638210

[48] Strand M Bulik CM. Trends in female authorship in research papers on eating disorders: 20-year bibliometric study. BJPsych Open. (2018) 4:39–46. 10.1192/bjo.2017.8 29467058 PMC6020273

[49] Aranda K. Feminism and nursing: an un/easy alliance of silences and absences. In:Lipscomb M, editor. Social Theory and Nursing. Heidelberg: Springer (2017). p. 115–32.

[50] Gunn V Muntaner C Ng E Villeneuve M Gea-Sanchez M Chung H. Gender equality policies, nursing professionalization, and the nursing workforce: a cross-sectional, time-series analysis of 22 countries, 2000–2015. Int J Nurs Stud. (2019) 99:103388. 10.1016/j.ijnurstu.2019.103388 31493758

[51] Cottingham MD. The missing and needed male nurse: discursive hybridization in professional nursing texts. Gen Work Org. (2019) 26:197–213. 10.1111/gwao.12333

[52] Manzi F. Are the processes underlying discrimination the same for women and men? A critical review of congruity models of gender discrimination. Front Psychol. (2019) 10:1–16. 10.3389/fpsyg.2019.00469 30894831 PMC6414465

[53] Hollup O. The impact of gender, culture, and sexuality on Mauritian nursing: Nursing as a non-gendered occupational identity or masculine field? Qualitative study. Int J Nurs Stud. (2014) 51:752–60. 10.1016/j.ijnurstu.2013.09.013 24144275

[54] McMurry TB. The image of male nurses and nursing leadership mobility. Nursing Forum. (2011) 46:22–8. 10.1111/j.1744-6198.2010.00206.x 21306392

[55] Sasa RI. Male nurse: a concept analysis. Nursing Forum. (2019) 54:593–600. 10.1111/nuf.12374 31463944

[56] Clifton A Higman J Stephenson J Navarro AR Welyczko N. The role of universities in attracting male students on to pre-registration nursing programmes: an electronic survey of UK higher education institutions. Nurse Educ Today. (2018) 71:111–5. 10.1016/j.nedt.2018.09.009 30278334

[57] Berkery E Tiernan S Morley M. The relationship between gender role stereotypes and requisite managerial characteristics: the case of nursing and midwifery professionals. J Nurs Manag. (2014) 22:707–19. 10.1111/j.1365-2834.2012.01459.x 23406476

[58] Charlesworth TES Banaji MR. Gender in science, technology, engineering, and mathematics: issues, causes, solutions. J Neurosci. (2019) 39:7228–243. 10.1523/JNEUROSCI.0475-18.2019 31371423 PMC6759027

[59] Carli LL Alawa L Lee YA Zhao B Kim E. Stereotypes about gender and science: women ≠ scientists. Psychol Women Q. (2016) 40:244–60. 10.1177/0361684315622645

[60] Moss-Racusin CA Dovidio JF Brescoll VL Graham MJ Handelsman J. Science faculty's subtle gender biases favor male students. Proc Natl Acad Sci U S A. (2012) 109:16474–9. 10.1073/pnas.1211286109 22988126 PMC3478626

[61] Pritlove C Juando-Prats C Ala-leppilampi K Parsons JA. The good, the bad, and the ugly of implicit bias. Lancet. (2019) 393:502–04. 10.1016/S0140-6736(18)32267-0 30739671

[62] Girod S Fassiotto M Grewal D Ku MC Sriram N Nosek BA. Reducing implicit gender leadership bias in academic medicine with an educational intervention. Acad Med. (2016) 91:1143–50. 10.1097/ACM.0000000000001099 26826068

[63] Raymond JL Goodman MB. Funders should evaluate projects, not people. Lancet. (2019) 393:494–5. 10.1016/S0140-6736(19)30280-6 30739667

[64] Sheltzer JM Smith JC. Elite male faculty in the life sciences employ fewer women. Proc Natl Acad Sci U S A. (2014) 111:10107–12. 10.1073/pnas.1403334111 24982167 PMC4104900

[65] Clayton-Hathway K Griffiths H Schutz S Humbert AL Mcilroy R. Gender and Nursing as a Profession: Valuing Nurses and Paying Them Their Worth. Royal College of London (2020). 1–90.

